# Hemopexin Modulates Expression of Complement Regulatory Proteins in Rat Glomeruli

**DOI:** 10.3390/cimb43020077

**Published:** 2021-09-07

**Authors:** Maria G. Detsika, Elias A. Lianos

**Affiliations:** 1 1st Department of Critical Care Medicine and Pulmonary Services, G. P. Livanou and M. Simou Laboratories, School of Medicine, National and Kapodistrian University of Athens, Evangelismos Hospital, 10675 Athens, Greece; 2 Veterans Affairs Medical Center and Virginia Tech., Carilion School of Medicine, 1970 Roanoke Blvd, Salem, VA 24153, USA; elias.lianos@va.gov

**Keywords:** decay accelerating factor (DAF), CD59, Crry, complement, hemopexin, hemin

## Abstract

In systemic hemolysis and in hematuric forms of kidney injury, the major heme scavenging protein, hemopexin (HPX), becomes depleted, and the glomerular microvasculature (glomeruli) is exposed to high concentrations of unbound heme, which, in addition to causing oxidative injury, can activate complement cascades; thus, compounding extent of injury. It is unknown whether unbound heme can also activate specific complement regulatory proteins that could defend against complement-dependent injury. Isolated rat glomeruli were incubated in media supplemented with HPX-deficient (HPX^−^) or HPX-containing (HPX^+^) sera as a means of achieving different degrees of heme partitioning between incubation media and glomerular cells. Expression of heme oxygenase (HO)-1 and of the complement activation inhibitors, decay-accelerating factor (DAF), CD59, and complement receptor-related gene Y (Crry), was assessed by western blot analysis. Expression of HO-1 and of the GPI-anchored DAF and CD59 proteins increased in isolated glomeruli incubated with HPX^−^ sera with no effect on Crry expression. Exogenous heme (hemin) did not further induce DAF but increased Crry expression. HPX modulates heme-mediated induction of complement activation controllers in glomeruli. This effect could be of translational relevance in glomerular injury associated with hematuria.

## 1. Introduction

In diseases associated with systemic hemolysis, including the hemolytic-uremic syndrome, immune-mediated hemolytic anemias, hemoglobinopathies, such as sickle cell disease and malaria, plasma levels of hemoglobin (Hb) increase, resulting in depletion of the Hb scavenging protein, haptoglobin. Subsequent oxidation of unbound Hb releases heme, which is rapidly bound by hemopexin (HPX). Saturation/depletion of HPX increases circulating levels of bioavailable (free) heme, which can cause oxidative organ stress/injury and inflammation [1].

Given that the normal renal glomerular microvasculature (glomeruli) filters ~ 180 L of plasma daily, it is not surprising that cells comprising glomeruli (endothelial, mesangial and podocytes) are subject to sustained exposure to circulating unbound heme when systemic hemolysis occurs. In addition to direct oxidative injury, unbound heme also activates complement via the alternative pathway thereby compounding extend of injury and inflammation mediated by heme alone. Thus, significant deposition of complement proteins indicative of complement activation was described in glomeruli of patients with hemolysis consequent to sickle cell disease [2]. It follows that scavenging of free heme by HPX could also mitigate heme-induced complement activation. This was shown in mice with drug-induced extensive hemolysis and concurrent complement activation. Intravenous HPX treatment prevented extent of complement activation and renal dysfunction [1].

Although complexing of free heme with HPX is expected to mitigate heme-induced cell injury and complement activation, HPX itself was reported to actually cause glomerular injury. Direct infusion of HPX into the renal microvasculature of rats results in proteinuria associated with ultrastructural podocyte (visceral glomerular epithelial cells) changes resembling human minimal change disease [2]. Moreover, direct exposure of cultured podocytes to HPX causes cytoskeletal remodeling of actin with loss of stress fibers and glycocalyx degradation [3]. These observations point to HPX, not only as a free heme scavenger, but also as a protein relevant in pathobiology of renal disease. Further evidence for this role comes from studies showing that HPX can be produced in glomeruli by cytokine-stimulated mesangial cells [4], and that plasma HPX activity is regulated by cytokine-stimulated mesangial cells [5]. Collectively, these observations highlight the complex role of heme:HPX binding in complement-mediated kidney injury.

Glomeruli are endowed with several cell-associated complement activation regulators that can minimize complement-mediated injury. Key among these complement regulatory proteins (CRP) in humans are decay-accelerating factor (DAF, CD55) and CD59 while in the rat a third CRP protein, complement receptor-related gene Y (Crry), was also identified. Both DAF and CD59 are membrane-bound via a glycosylphosphatidylinositol (GPI) anchor and control either early (DAF) or terminal (CD59) stages of complement activation. We previously reported that free heme induces DAF in isolated rat glomeruli via a heme oxygenase (HO)-1 dependent mechanism [3]. The translational relevance of this observation becomes apparent in view of evidence that free heme activates the alternative complement pathway in normal human serum, releasing C3a, C5a, and sC5b9, and also causes C3 and C5b-9 binding in cultured cells [6].

In the experiments assessing effect of heme on glomerular DAF induction, heme (hemin) was directly introduced in glomeruli incubated with media containing normal (HPX-containing) serum. As such, the modulatory effect of HPX present in serum on heme-mediated glomerular DAF or other CRP induction could not be assessed. The present study addresses this question using glomeruli incubated with media containing either normal (HPX-containing) or HPX-deficient serum obtained from HPX-deficient mice generated as previously described to achieve high tissue levels of unbound heme [4,5].

## 2. Materials and Methods

### 2.1. Reagents

Rat anti-DAF antibody clone RDIII-7 (catalogue number: HM3035) was purchased from Hycult (Hycult, Uden, The Netherlands) as were anti-Crry clone TLD-1C11 (catalogue number: HM3032) and anti-CD59 clone TH9 (catalogue number: HM3037). Nrf2 antibody was purchased form Abcam (Abcam, Cambridge, UK). Anti-β-actin antibody was purchased from Sigma (Sigma-Aldrich, St Louis, MO, USA) and anti-GAPDH antibody from Cell Signaling (Cell Signaling, Danvers, MA, USA). HPX-deficient (HPX¯) serum was a kind gift from Prof E. Tolosano and originated from HPX-null mice generated as previously described [7]. Successful HPX depletion in HPX-null mice was demonstrated by Northern blot analysis on total RNA extracted from the liver of wild type, heterozygous, and homozygous littermates. HPX mRNA was absent in the liver of HPX^−/−^ mice and was about half normal in HPX^+/−^ mice, indicating that there was no compensation for the reduced gene dosage of Hx in heterozygous mice. Absence of HPX protein in sera of HPX-null mice was demonstrated by Western blot analysis. Phenotypically, there was no effect on plasma levels of iron, bilirubin, albumin, or blood cell lineages in HPX-null mice. Moreover, histologic evaluation of liver, kidney, heart, brain, spleen, and bone marrow revealed no lesions while staining of these tissues for iron revealed no increase in iron deposition. However, following drug-induced systemic hemolysis or intravenous heme treatment, HPX-deficient mice developed pro-longed hemoglobinuria with high kidney iron content and degree of lipid peroxidation compared to control mice [7,8]. Hemopexin-containing (HPX^+^) serum (control) was obtained from whole blood of wild-type mice. Sera from mouse (HPX^+^) were obtained from Sigma Aldrich (St. Louis, MO, USA). Hemin was obtained from Sigma Aldrich.

### 2.2. Animals

Six-week-old adult male Sprague–Dawley rats, 250 g in body weight, were employed in this study. Animals were reared in accordance to the European Union Directive for care and use of laboratory animals and all procedures were approved by the Hellenic Veterinary Administration and the ethics committee of ‘Evangelismos’ Hospital.

### 2.3. Isolation of Glomeruli and Incubations

Glomeruli were isolated from kidneys of wild type (WT) rats by an established differential sieving method [9]. Following isolation, glomeruli were plated in six-well plates and incubated in media (Dulbecco’s Modified Eagle Media, DMEM) containing defined volumes (%v/v) of HPX^−^ or HPX^+^ serum for 18 h in the presence or absence of defined heme (hemin) concentrations. HPX^−^ serum, obtained from HPX-deficient mice and mouse sera obtained from WT mice (Sigma-Aldrich) was used as HPX^+^ serum for controls in all incubations. Upon completion of incubations, total glomerular protein was extracted using a lysis buffer (150 mM NaCl, 50 mM Tris pH 8.0, and 1% Triton X containing a protease inhibitors cocktail) as described previously [10]. Concentration of protein samples was determined by the Bradford assay.

### 2.4. Western Blotting

Tissue protein lysates were resolved by SDS-PAGE, transferred onto polyvinylidene difluoride (PVDF) membrane, and probed with primary antibodies overnight at the following concentrations: 1:2000 for the anti-DAF, 1:1000 for the anti-Crry and 1:100 for the anti-CD59, anti-HO-1, and anti-Nrf2 antibodies. Western blots were run under non-reducing conditions for DAF, Crry, and CD59, according to the suppliers’ instructions. Western blots for all other primary antibodies were run under reducing conditions. Incubations with primary antibodies were performed overnight at 4 °C followed by three 10 min washes with phosphate buffered saline tween (PBST) for anti-DAF, anti-Crry, and anti-CD59 antibodies or tris-buffered saline tween (TBST) for HO-1 and Nrf2 antibodies, and two-hour incubations with secondary antibodies at room temperature. Six-minute washes were repeated five times after secondary antibody incubations prior to membrane visualization by enhanced chemiluminescence (ECL) reagent obtained from Santa Cruz (Santa Cruz, Dallas, TX, USA). Equal protein loading was determined by probing for β-actin or GAPDH. Densitometric analysis was performed using the GelPro analyzer software (GelPro analyzer version 3.1. Media Cybernetics, Silver Spring, MD, USA) by obtaining the ratios of the appropriate band over β-actin or GAPDH respectively.

### 2.5. Statistical Analyses

Values are expressed as mean ± SE (standard error). Statistical analyses were performed with analysis of variance (ANOVA) for more than two group comparisons. Post-hoc analysis was performed with the least significant test. A *p* value < 0.05 was chosen as statistically significant.

## 3. Results

### 3.1. CD59 and Crry Expression in Isolated Glomeruli

**Figure 1:**
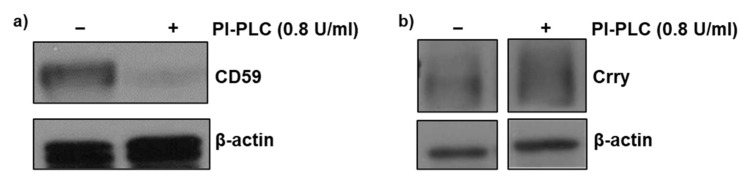
Detection of glomerular CD59 and Crry. Glomeruli were incubated with phosphatidylinositol-specific phospholipase C (PI-PLC) (0.8 U/mL) for 90 min to confirm the presence of GPI-anchored CD59 protein in isolated glomeruli and presence of membrane bound Crry. Total protein lysates were analyzed by Western blot for (**a**) CD59 and (**b**) Crry protein. β-actin was used as a loading control.

Initial experiments were carried out to assess whether expression of GPI-anchored CD59 was preserved following the glomerular isolation procedure. Isolated glomeruli were incubated in the presence and absence of phosphatidyl-specific phospholipase C (PI-PLC) at a concentration (0.8 U/mL) sufficient to cleave the GPI anchor. Western blot analysis of glomerular lysates revealed almost complete absence of CD59 in the presence of PI-PLC (Figure 1a) indicating that isolation procedure had no major effect on GPI-anchored CD59. We previously reported a similar observation for GPI-anchored DAF expression in isolated glomeruli [11]. Therefore, these experiments were not repeated. In contrast, PI-PLC treatment had no effect on the non-GPI-anchored protein, Crry (Figure 1b).

### 3.2. HPX^−^ Deficient Serum Increases Glomerular HO-1 Expression

**Figure 2:**
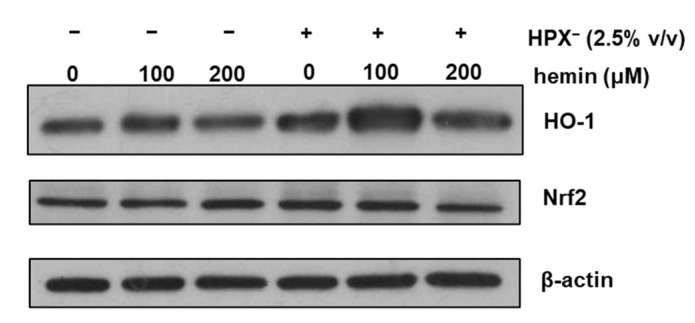
Isolated glomeruli were incubated with serum-free media or media supplemented with 2.5% HPX^−^ serum for 18 h in the presence of increasing concentrations of hemin. Total protein lysates were analyzed by western blotting for HO-1 protein and Nrf2 protein. β-actin was used as loading control.

To determine whether HPX^−^ deficient serum increases glomerular HO-1 expression, isolated glomeruli were incubated in serum-free media or media supplemented with 2.5% (*v*/*v*) HPX¯ serum for 18 h in the presence or absence of exogenous heme (hemin). As shown in Figure 2, HO-1 protein expression was increased in glomeruli incubated with media containing HPX^−^ serum compared to incubations in the absence of serum (Western blot lane 4 vs. lane 1). HO-1 induction in response to exogenous hemin was augmented in glomeruli incubated with HPX^−^ serum while a similar expression pattern to HO-1 was observed for nuclear factor erythroid 2-related factor 2 (Nrf2).

### 3.3. Effect of HPX^−^ Deficient Serum on Glomerular DAF, Crry, and CD59

**Figure 3:**
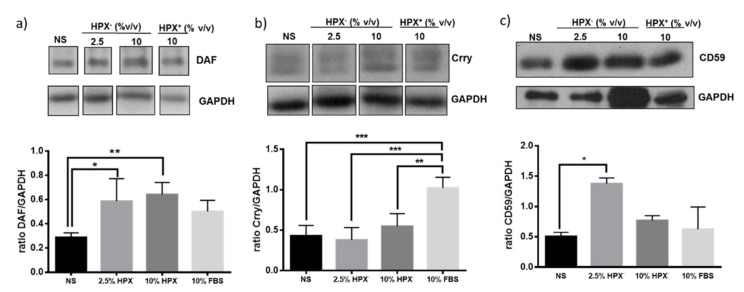
Glomeruli were incubated with media containing no serum (NS), various amounts of HPX^−^ serum (2.5%, 10% v/v) or with HPX^+^ serum (10%) for 18 h. Total protein lysates were analyzed by western blotting for: (**a**) DAF, (**b**) Crry, and (**c**) CD59 glomerular expression. Representative western blot from three independent experiments is shown. Data are expressed as means ± SEM. * *p* < 0.05; ** *p* < 0.01, *** *p* < 0.001 (ANOVA and post hoc analysis by the least significant difference test). GAPDH was used as loading control. Bands separated by gaps are chosen from the original western blot (Figure S1). For Crry western blot, membrane for DAF blot was incubated in mild stripping buffer (overnight) and re-probed with Crry antibody solution.

Isolated glomeruli were incubated in media supplemented with HPX-containing (HPX^+^, 10% *v*/*v*) serum, HPX deficient (HPX^−^, 2.5 or 10% *v*/*v*) serum or with serum-free media. Statistically significant induction of Crry but not DAF or CD59 occurred in response to 10% HPX^+^ serum compared to incubations with serum-free media (Figure 3). There was a significant increase in DAF and CD59, but not in Crry protein expression in glomeruli incubated with media supplemented with 2.5 and 10% HPX^−^ serum, indicating a differential effect on GPI-anchored CRPs.

### 3.4. Effect of Heme on Glomerular DAF and Crry Expression

**Figure 4:**
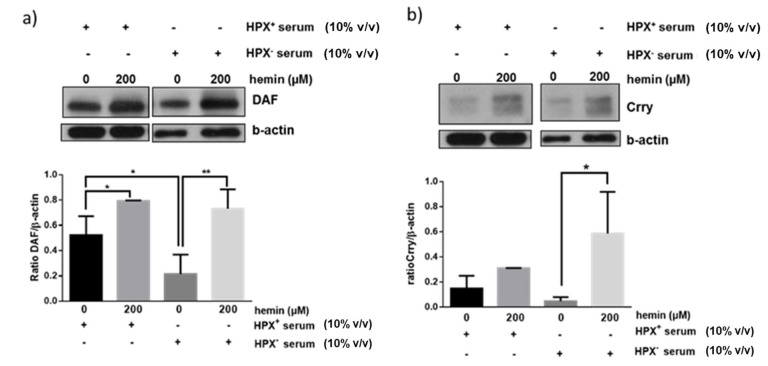
Isolated glomeruli were incubated with 10% HPX^−^ or 10% HPX^+^ serum in the presence of exogenous hemin (200 μM) for 18 h. Total protein lysates were analyzed by western blotting for (**a**) DAF and (**b**) Crry protein. Representative western blots from three independent experiments are show. Densitometric analyses of data are expressed as means ± SEM.* *p* < 0.05; ** *p* < 0.01; (ANOVA and post hoc analysis by the least significant difference test). β-actin was used as loading control. Original western blot shown in Figure S2. For Crry western blot, membrane for DAF blot was incubated in mild stripping buffer (overnight) and re-probed with Crry antibody solution.

We next assessed whether exogenous heme (hemin) could modulate effect of HPX⁺ or HPX^−^ sera on glomerular expression of CRPs. Glomeruli were incubated for 18 h with hemin in the presence of HPX^+^ (10% *v*/*v*) or HPX^−^ (10% *v*/*v*) serum in the presence of hemin concentrations (200 μM) likely to be encountered in serum during systemic hemolysis and previously shown to induce DAF [11]. As shown in Figure 4a, a similar degree of DAF induction in response to 200 μΜ hemin was observed in the presence of both HPX^+^ and HPX^−^ deficient serum. On the contrary, Crry induction was augmented in presence of HPX^−^ serum (Figure 4b).

## 4. Discussion

Hemoglobin and heme continuously leak from red blood cells in plasma and tissues in what is known as trivial hemolysis [12]. In diseases associated with systemic hemolysis, such as paroxysmal nocturnal hemoglobinuria and hemolytic uremic syndrome, or in aggressive forms of glomerular injury in which hemoglobinuria owing to erythrocyte injury/lysis within glomerular capillaries is a prominent feature, cells comprising the glomerular microvasculature (endothelial cells, mesangial cells, and podocytes) are exposed to high concentrations of free/labile heme released from of hemoglobin (Hb). Exposure to free heme markedly increases when heme-binding plasma proteins (Hemopexin, Albumin, a1-microglobulin, a1-antitrypsin) become saturated/depleted. Of the heme-binding proteins (HBPs), HPX and albumin are best known for their role in minimizing cellular uptake of heme and consequent toxicity. HPX binds heme with the highest binding affinity of all known HBPs and transports it to the liver for degradation [13] whereas albumin, due to its abundance, may act as a transient heme-binding protein and transfers heme to Hx [14].

Although concentrations of bioavailable free heme attained within glomerular capillaries (glomeruli) are unknown, circulating free heme in systemic hemolytic diseases can reach concentrations higher than 150 µM [15]. Even though heme:HPX complexes form readily, filtration of these complexes by glomeruli is impaired owing to their size [16], thus facilitating their delivery and internalization/endocytosis by intrinsic glomerular cells via specific receptors, particularly the low-density lipoprotein receptor-related protein, also known as CD91 receptor [17]. Heme uptake in the form of heme:HPX complexes was shown to increase HO-1 expression and this HO stimulatory pathway is different from expression increase by free heme, which can also be imported into cells directly by heme importers [9].

In neutral solutions and in the presence of oxygen heme is rapidly converted to hemin [10]. Previous experiments assessing effect of hemin on HO-1 induction in isolated glomeruli employed hemin at concentrations likely to be attained in circulation following systemic hemolysis. These experiments demonstrated that, in addition to HO-1, hemin also upregulates glomerular expression of the cell-associated GPI-anchored CRP, DAF [11]. However, in those experiments, glomeruli were incubated with hemin in the presence of normal (HPX^±^) serum and, therefore, the high amphipathicity of hemin and its high affinity and complexing with HBPs was not taken into consideration.

In the present study, glomeruli were incubated with media containing either normal (HPX^+^) or HPX^−^ serum to determine extent to which presence of HPX modulates baseline and heme-induced expression of HO-1 and of the CRPs, DAF, CD59, and Crry. Both DAF and CD59 are GPI-anchored to cell membranes. DAF accelerates the dissociation of C3 and/or C5 convertases while CD55 prevents assembly of the membrane attach complex, C5b-9, on cell membranes [18,19]. Changes in expression of the non-GPI anchored CRP, Crry, which is uniquely expressed in rodents and combines the functions of DAF and membrane co-factor protein (MCP), were also assessed.

In previous studies [11], we demonstrated that expression of GPI-anchored DAF is preserved following isolation of rat glomeruli and it is induced by heme (hemin). As shown in Figure 1, expression of GPI-anchored CD59 is also preserved as incubation with PI-PLC resulted in total loss of CD59 due to cleavage of the GPI-anchor. As expected, PI-PLC treatment had no effect on the non-GPI-anchored Crry. In glomeruli incubated with media containing 2.5% HPX^−^ serum, HO-1 expression increased while that in response to exogenous heme was augmented (Figure 2). This is in agreement with previous studies demonstrating augmentation of HO-1 expression in various tissues of HPX-deficient mice following direct tissue exposure to exogenous heme given intravenously at high doses of 70 µM/Kg sufficient to cause increased organ heme content and lipid peroxidation [8]. The augmented glomerular HO-1 expression observed in glomeruli incubated with HPX^−^ serum in the presence of exogenous hemin can be attributed to the increased heme content achieved.

Expression of DAF and CD55, but not that of Crry protein, also increased in glomeruli incubated with media supplemented with 2.5% HPX^−^ serum (Figure 3). Media containing 10% HPX^−^ serum had no further effect on DAF or CD59 protein indicating that 2.5% HPX^−^ serum was sufficient to maximize induction of these CRPs. In media containing 10% HPX^+^ serum, the increase DAF or CD59 protein was not significant (Figure 3a,c). However, Crry protein level increased significantly (Figure 3b). Taken together, these results indicate that serum HPX differentially modulates expression of glomerular CRPs.

The mechanisms underlying modulation of glomerular CRPs by HPX is unclear and requires analysis of potential CRP inducers present in HPX-deficient vs. HPX^+^ serum as well as assessment of extent to which heme:HPX complex formation and internalization by glomerular cells is necessary and sufficient for heme delivery and induction of HO-1 or CRPs via this pathway. For example, in media supplemented with HPX-deficient serum formation and internalization of unbound heme:HPX complexes would be minimal or absent. Therefore, increase in expression of HO-1 (Figure 2), of DAF (Figure 3a) and CD59 (Figure 3c) likely occurred in a manner independent of heme:HPX complex formation and internalization, a likely mechanism being direct heme internalization by heme importers. Further, glomerular DAF induction in response to exogenous heme occurred to a similar extent in incubations with HPX-containing or HPX-deficient sera (Figure 4a) indicating that heme:HPX complex formation/internalization did not play a major role. In contrast, Crry induction in response to exogenous heme was augmented in incubations with HPX-deficient serum (Figure 4b) indicating that unbound heme present in this serum was not of sufficient concentration to induce Crry and further supporting the argument that serum HPX differentially modulates expression of glomerular CRPs.

Analysis of HPX-deficient serum for presence of potential CPR inducers could point to mechanisms other that heme:HPX complex formation. Such analyses would require proteomic/genomic methods to identify genes transcriptionally regulated by lack of HPX. In one such analysis, genes highly probable to be functionally related to HPX were identified in HPX-deficient mice and include the Ras suppressor-1 (Rsu1), originally identified as a suppressor of Ras-dependent oncogenic transformation, and the cytokine MdK, which was shown to regulate leukocyte trafficking and adhesion [20]. Assessment of CRPs in glomeruli of hemopexin-deficient animals could also provide mechanistic insights. HPX deficient mice subjected to drug-induced systemic hemolysis resulting in HPX saturation/depletion develop hemoglobinuria and severe kidney injury involving primarily proximal tubules [7]. However, glomerular expression of CRPs was not assessed in this model.

These observations are of translational relevance in hemolytic diseases and in kidney diseases associated with hematuria of glomerular origin as incubation of glomeruli with HPX deficient serum ex vivo resembles exposure to free heme in hemolytic diseases causing depletion of circulating HPX. Free heme activates complement [6] while resident glomerular cells, particularly podocytes, are vulnerable to both heme-mediated toxicity and complement activation [21]. In this regard, both complement factor 3 (C3) and membrane attack complex (C5b-9) deposits are found in glomeruli of patients with sickle cell disease, which is characterized by episodes of intravascular hemolysis resulting in increased unbound heme concentrations and HPX depletion/deficiency in the circulation [22]. The demonstration that HPX deficiency can increase glomerular expression of DAF and CD59 indicates that, even though unbound heme can cause complement activation, it can also activate specific complement regulatory proteins that could defend against complement-dependent injury.

## Supplementary Materials

The following are available online at https://www.mdpi.com/article/10.3390/cimb43020077/s1.
