# Supplementary material for: Hemopexin Modulates Expression of Complement Regulatory Proteins in Rat Glomeruli
Source: Curr Issues Mol Biol. 2021 Sep 7;43(2):1081–9. doi: 10.3390/cimb43020077 (PMC8928991; doi:10.3390/cimb43020077)
Supplement: Supplementary file 1 [file cimb-43-00077-s001.zip › cimb-1324928-supplementary/cimb-1324928-supplement- final.pptx]

## Slide 1
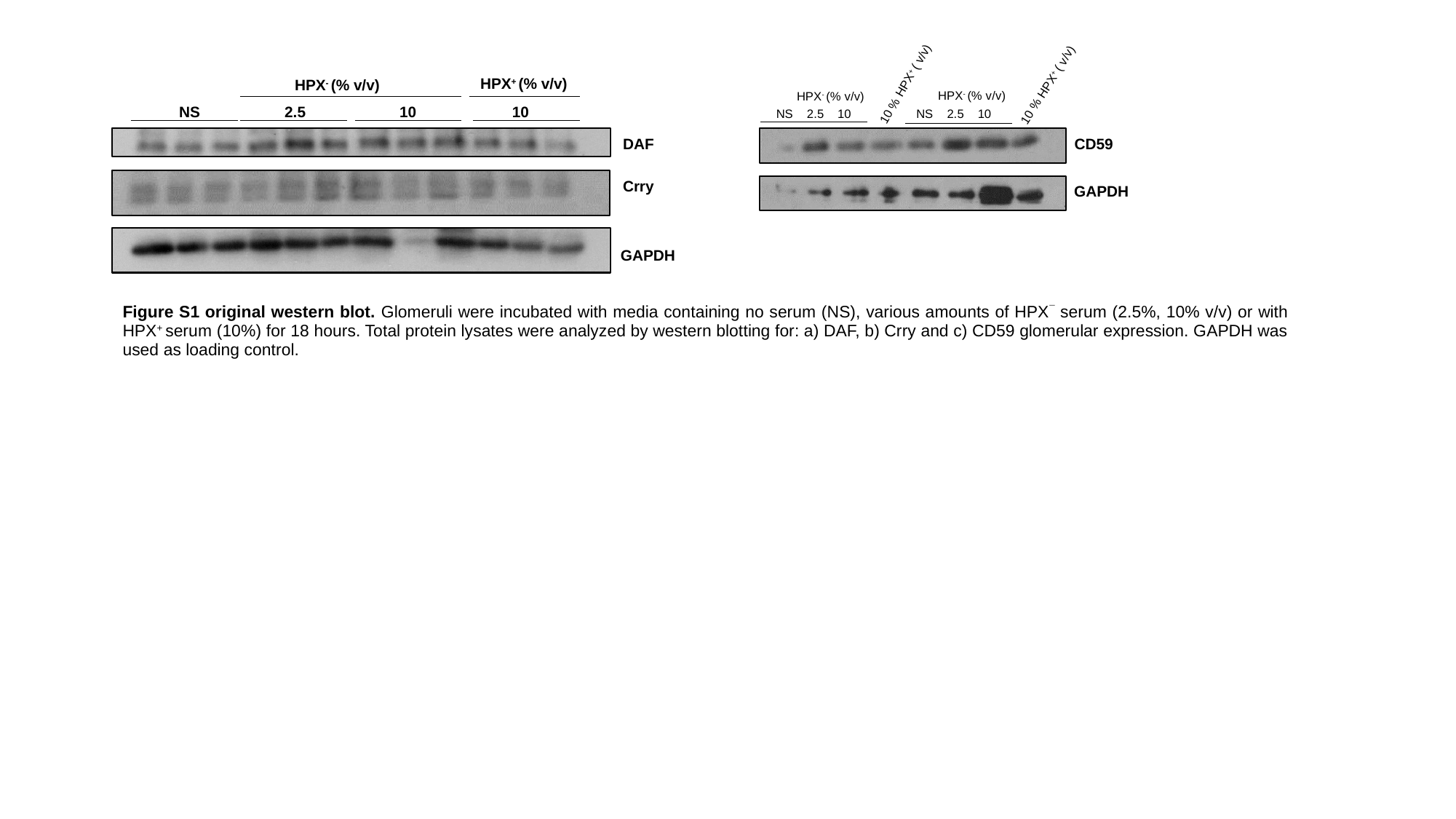

10 % HPX+ ( v/v)
10 % HPX+ ( v/v)
HPX- (% v/v)
HPX- (% v/v)
NS 2.5 10
NS 2.5 10
CD59
GAPDH
HPX+ (% v/v)
HPX- (% v/v)
NS
2.5
10
10
DAF
Crry
GAPDH
Figure S1 original western blot. Glomeruli were incubated with media containing no serum (NS), various amounts of HPX¯ serum (2.5%, 10% v/v) or with HPX+ serum (10%) for 18 hours. Total protein lysates were analyzed by western blotting for: a) DAF, b) Crry and c) CD59 glomerular expression. GAPDH was used as loading control.

## Slide 2
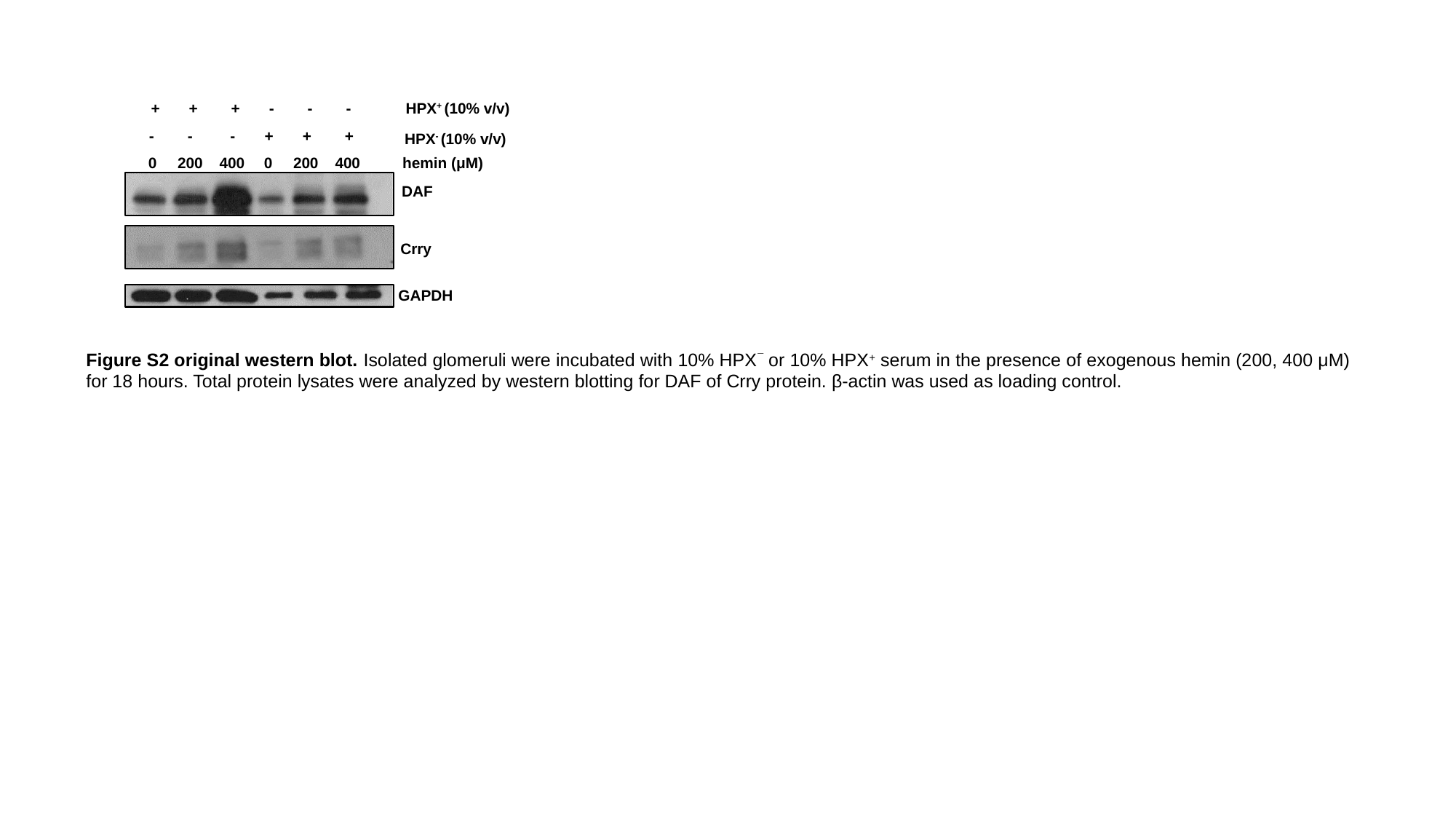

HPX+ (10% v/v)
+ + + - - -
- - - + + +
HPX- (10% v/v)
0 200 400
0 200 400
hemin (μΜ)
DAF
Crry
GAPDH
Figure S2 original western blot. Isolated glomeruli were incubated with 10% HPX¯ or 10% HPX+ serum in the presence of exogenous hemin (200, 400 μM) for 18 hours. Total protein lysates were analyzed by western blotting for DAF of Crry protein. β-actin was used as loading control.
